# K-Sign in retrocaecal appendicitis: a case series

**DOI:** 10.1186/1757-1626-2-157

**Published:** 2009-10-19

**Authors:** Imtiaz Wani

**Affiliations:** 1 Department of Pediatric Surgery, Sheri-Kashmir Institute of Medical Sciences, Srinagar, Kashmir, India; 2 Department of General Surgery, Sheri-Kashmir Institute of Medical Sciences, Srinagar, Kashmir, India; 3 Department of Accident and emergency, Sheri-Kashmir Institute of Medical Sciences, Srinagar, Kashmir, India

## Abstract

**Background:**

Variations in position of the vermiform appendix considerably changes clinical findings. Retrocaecal appendicitis presents with slightly different clinical features from those of classical appendicitis associated with a normally sited appendix. K-sign looks for the presence of tenderness on posterior abdominal wall in the retrocaecal and paracolic appendicitis. This is the first case report of this kind in the literature. The K-sign has been named, as a mark of respect, after the region of origin of this sign, Kashmir, so called as "Kashmir Sign". The sign being present in view of inflamed appendix crossing above its non palpable position above iliac crest on the posterior abdominal wall and the tenderness is by irritation of posterior peritoneum

**Case presentation:**

The author is reporting a case series of four patients in whom a K-sign, a clinical sign, was elicited and found positive on the posterior abdominal wall for presence of tenderness in a specific area bound by the 12^th ^rib superiorly, spine medially, lateral margin of posterior abdominal wall laterally and iliac crest inferiorly and was found to be present in three retrocaecal and one paracolic appendicitis. Each case had tenderness in this specific area on posterior abdominal wall. All had appendectomy and having histopathological evidence of appendicitis.

**Conclusion:**

K-sign can be useful in diagnosis of retrocaecal and paracolic appendicitis. Significance of K-sign being in view of difficulty in diagnosis of retrocaecal appendicitis and its subsequent complications.

## Background

Retrocaecal appendix forms 65% of position of appendix and the retrocaecal appendicitis has several atypical presentations with a few specific signs. There is often limited systemic upset and no progression to affect the general peritoneal cavity [1,2]. In the early course of the retrocaecal appendicitis, there may not be enough inflammation to cause this tenderness, although it is unusual for patients to present at early in the course of their illness. In appendicitis other than the retrocaecal type, tenderness is due to inflammation of the serosa of the appendix and the overlying parietal peritoneum but because of its position, tenderness in right lower abdomen in retrocaecal appendicitis is not always present and may present difficulty in diagnosis. It is to be stressed that diagnostic accuracy of appendicitis increases with a more thorough knowledge of the signs and symptoms of acute appendicitis and a constant awareness of its possible presence which gives importance of K-sign in appendicitis to a clinician[3,4].

## Methods of eliciting K-sign

K-sign is elicited in the left lateral position by percussion of posterior abdominal wall in an area bound by spine medially,12^th ^rib superiorly, iliac crest inferiorly and lateral margin of posterior abdominal wall laterally, and recording an area of tenderness. Percussion is started at lateral margin of posterior abdominal wall, from 12^th ^rib towards iliac crest in an above to downward direction and whole area is palpated for presence of tenderness, each time starting from 12^th ^rib and the area of tenderness recorded. Percussion for presence of K-sign can be also done by palpation in lateral to medial direction from lateral margin of posterior abdominal wall towards the spine and whole area is palpated, each time starting from lateral margin of posterior abdominal wall and the specific area of tenderness is noted.

## Case presentations

### Case report 1

A 12 year old Kashmiri boy of Indian ethinicity presented with pain right lower abdomen and fever of 12 hours duration. There was history of nausea and anorexia. Fever of 99 degrees Farhereneit was recorded. Per abdominal examination revealed tenderness on deep palpation in right iliac fossa. K-sign was found to be positive; an area of tenderness was found present in between middle of psoas muscle and an adjacent area lateral to psoas muscle extending superiorly from iliac crest. Psoas sign was found to be positive. X-ray abdomen was unremarkable. Abdominal sonography findings being a non compressible structure in right lower quadrant suggestive of inflamed appendix. Patient had appendectomy and the 11. 4 centimeters long inflamed appendix being retrocaecal in position was seen. Contents in appendix was pus. Post operative period and follow up being uneventful

### Case report 2

13 year old Kashmiri boy of Indian ethinicity presented with pain right flank, fever, vomiting and anorexia of 2 days duration. General physical examination revealed pulse of 96/min. and fever of 99.5 degrees Fahrenheit. Rebound tenderness and Rovsing's sign was present on per abdominal examination. Psoas sign was negative. K-sign was elicited and was positive present in an area extending from lateral margin of psoas muscle to flank extending from iliac crest. Abdominal sonography was inconclusive. Patient had appendectomy having 10.6 centimeters long inflamed paracoilc appendix with fecalith inside. Histopathology was consistent with appendicitis Follow up was uneventful

### Case report 3

A 9 year old Kashmiri girl of Indian ethinicity presented with epigastric pain and vomiting of 4 hours duration. She was kept under observation and after duration of 6 hours there was shift of pain to right iliac fossa and fever of 99 degrees Farhereneit was recorded. She had pulse of 98/min and B.P of 100/70 mm. Hg. Abdominal examination revealed no tenderness in right iliac fossa. Psoas sign was found to be positive. A K-sign was found to be positive in small area on psoas muscle and adjacent posterior abdominal wall with superior extension from iliac crest. On X-ray abdomen there was localized ileus in right lower quadrant. Abdominal sonography showed a non compressible structure suggestive of inflamed appendix. Patient had appendectomy and an 8.4 centimeter long retrocaecal appendix was seen containing multiple fecalith inside. Patient was discharged on 4^th ^postoperative day and had uneventful follow up.

### Case report 4

A 34 year old Kashmiri male of Indian ethnicity presented with pain right upper abdomen, vomiting and fever of 2 day's duration. General physical revealed dehydrated look, pulse of 96/min, B.P of 120/80 mm Hg. and temperature of 100 degree Fahrenheit. Hemoglobin of 12 g./dl. TLC of 14,000/mm^3 ^was present. Abdominal examination revealed tenderness in right upper and middle quadrant, the right iliac fossa was free. Psoas sign was positive. K-sign was found positive extending to 12^th ^rib along lateral margin of psoas muscle from the iliac crest. X-ray abdomen showed localised ileus in right upper area of abdomen. Ultrasound abdomen was suggestive of acute appendicitis with periappendiceal collection. Patient had appendectomy and peroperative findings revealed retorcaecal, subhepatic 17.4 cm long appendix with surrounding pus present. Histopathology documented appendicitis and had uneventful postoperative as well as follow up period.

## Discussion

Retrocaecal appendicitis lacks distinctive clinical pattern and has been theorized to follow a more insidious course than other anatomic variants[5,6]. Like all other positions of the appendix, retrocaecal appendicitis can be reliably diagnosed clinically with history taking and physical examination combined with the laboratory investigations. This retroperitoneal appendicitis is associated with a high incidence of retroperitoneal inflammatory changes which can permeate retroperitoneal fasciae and fatty tissue. For all types of appendicitis, tenderness over the site of the appendix is the sine qua non of appendicitis; patients with retrocaecal appendicitis have tenderness in the area of the appendix; provided that the area can be palpated. In retrocaecal appendicitis it is difficult to found tenderness on palpation in the right iliac region. Even deep pressure in the right lower quadrant may fail to elicit tenderness the reason being that the caecum, distended with gas, prevents the pressure exerted by the palpating hand from reaching the inflamed appendix, so it has been called as ' silent appendicitis'. Patients with a retrocaecal appendix may experience some mild right-sided or right flank tenderness. Long retro-colic inflamed appendix may cause confusions with cholecystitis (sub hepatic). Irritation of the posterior peritoneum may occur in retrocaecal or paracolic appendicitis and give psoas sign positive.

Mc Burney's point corresponds on right lateral line just 1.2-2.5 centimeters below the junction of intersection of right lateral plane and transtubercular plane, iliac bone along with its attachments, does not permit for **eliciting **tenderness at Mc Burney's point from back. Normal length of appendix ranges from 2-20 centimeters, with an average length of 9 centimeters and consequently in retrocaecal and paracolic type it will be raised above illac crest, tenderness will be elicited in that situation on an area of contact of inflamed appendix with posterior parietal peritoneum and the area will be corresponding area of posterior abdominal wall. Area of tenderness will depend upon length, intrinsic position of appendix, portion of appendix with inflammation, direction of appendix, presence of fibrosis, any kinking or adhesions as well as on the position of caecum. In nutshell, this sign is due to irritation by inflamed appendix of overlying posterior parietal peritoneum as in psoas sign and this sign simulates for eliciting tenderness in appendicitis in right iliac fossa. In our each case, appendix was long enough to cross above iliac crest and being retrocaecal and paracolic in position, sign was found to be positive. Further it has been rightly said that the meaning of sign in appendicits is never complete without reference to the topographical anatomy of the little organ and the situation of an acutely inflamed appendix can near always be determined by pressure method which is in support of this sign[7].

The significance of this sign in the retrocaecal and paracolic variety of appendix will lead to early diagnosis as these positions of appendix have the more common chances of gangrenous complication because their blood supply more prone to kinked be more liable to inflammation when fixed retro-caecally [8] Drawback with this sign is that it cannot be elicited in obese patients since the retrocaecal appendix is so well encased in retroperitoneal fat that it cannot be easily palpated and, those having very short retrocaecal or paracolic appendix. K-sign can be positive in other retroperitoneal pathologies like renal colic, ureteric colic and psoas abscess etc. K-sign can be useful sign in retrocaecal and paracolic appendicitis. Sometimes when meager symptoms and are signs present, this sign can be contributory in diagnosis.

## Competing interests

The author declares that he has no competing interests.

## Authors' contributions

IW: took acquisition of data, compilation of relevant literature, formatting, revision, drafted the preliminary and final manuscript.

## Consent

Written informed consent was obtained from the patients or their parents for publication of this case reports. A copy of the written consents is available for review by the Editor-in-Chief of this journal
